# NDM-5-Producing *Escherichia coli* Co-Harboring *mcr-1* Gene in Companion Animals in China

**DOI:** 10.3390/ani12101310

**Published:** 2022-05-20

**Authors:** Xu Kuang, Runshi Yang, Xinqing Ye, Jian Sun, Xiaoping Liao, Yahong Liu, Yang Yu

**Affiliations:** 1 National Risk Assessment Laboratory for Antimicrobial Resistance of Animal Original Bacteria, South China Agricultural University, Guangzhou 510642, China; kx@stu.scau.edu.cn (X.K.); promish@163.com (X.Y.); jiansun@scau.edu.cn (J.S.); xpliao@scau.edu.cn (X.L.); lyh@scau.edu.cn (Y.L.); 2 Guangdong Provincial Key Laboratory of Veterinary Pharmaceutics Development and Safety Evaluation, South China Agricultural University, Guangzhou 510642, China; 3 National Reference Laboratory of Veterinary Drug Residues, College of Veterinary Medicine, South China Agricultural University, Guangzhou 510642, China; 4 Guangdong Provincial Key Laboratory of Microbial Safety and Health, State Key Laboratory of Applied Microbiology Southern China, Institute of Microbiology, Guangdong Academy of Sciences, Guangzhou 510070, China; yangrunshi1@gmail.com; 5 Guangdong Laboratory for Lingnan Modern Agriculture, Guangzhou 510642, China

**Keywords:** NDM-5, MCR-1, companion animals

## Abstract

**Simple Summary:**

This is a study related to NDM-5-producing *E. coli* in companion animals in China. Notably, an *E. coli* isolate possessing both *bla*_NDM-5_-bearing plasmid and *mcr-1*-bearing plasmid was identified in a dog from the same veterinary clinic, where we previously found the mobile IncX3–X4 hybrid plasmid encoding both *bla*_NDM-5_ and *mcr-1* in a cat. This observation highlights the diversity of *bla*_NDM-5_- and *mcr-1*-harboring plasmids and the transferability of such resistant pathogens from companion animals to humans. Given that colistin is the last-resort antibiotic for treating human infections caused by carbapenem-resistant *Enterobacteriaceae*, the co-transfer of resistance to both antibiotics may seriously compromise the effectiveness of clinical therapy.

**Abstract:**

Carbapenem and colistin are important antibiotics for the treatment of infections caused by multidrug-resistant Gram-negative pathogens. Here, we isolated the *bla*_NDM-5_-harboring *Escherichia coli* in companion animals in healthy or diseased companion animals from veterinary clinics in six cities in China from July to November 2016. A total of 129 rectal swabs of healthy or diseased dogs and cats were collected from veterinary clinics in six different cities in China, and the isolates were subjected to carbapenem and colistin susceptibility testing. Resistance genes were confirmed using PCR. Conjugation experiments were conducted to determine the transferability of antibiotic resistance genes (ARGs) in the strains. The isolated rate of *bla*_NDM-5_-harboring *E. coli* strains was 3.88% (five strains). These five strains were multidrug resistant to at least three antibiotics and corresponded to four sequence types including ST101. The *bla*_NDM-5_ gene was located on 46 kb IncX3 plasmids in these five strains, and the genetic contexts were shared and were nearly identical to the *K. pneumoniae* plasmid pNDM5-IncX3 from China. In addition, one strain (CQ6-1) co-harbored *bla*_NDM-5_-encoding-IncX3 plasmid along with a *mcr-1*-encoding-IncX4 plasmid, and their corresponding genetic environments were identical to the *bla*_NDM-5_-IncX3 and *mcr-1*-IncX4 hybrid plasmid reported previously from the same area and from the same clinic. The results indicated that the similar genetic contexts were shared between these isolates from companion animals, and the IncX3-type plasmids played a key role in the spread of *bla*_NDM-5_ among these bacteria.

## 1. Introduction

Antimicrobial resistance (AMR) within bacteria is a growing public health threat of concern because of the overuse of antibiotics. Notably, carbapenem resistance has spread globally, and carbapenem-resistant *Enterobacteriaceae* (CRE) are a current global health crisis. Carbapenems are broad-spectrum β-lactam antibiotics and are considered as a last resort for multidrug-resistant (MDR) bacterial infections. In 2013, the United States Centers for Disease Control and Prevention (CDC) proclaimed that CRE infections are one of the top three most urgent threats to public health due to antimicrobial resistance. In particular, the New Delhi metallo-β-lactamases (NDM), capable of hydrolyzing all penicillins, cephalosporins and carbapenems, is now present in more than 70 countries and has been identified on the bacterial chromosome as well as plasmids [1,2,3,4]. The NDM-5 variant differs from the prototype NDM-1 by the possession of two amino acid substitutions (Val88Leu and Met154Leu) that directly confer an increased level of resistance to carbapenems and to the broad-spectrum cephalosporins [5]. NDM-5 was first identified in the United Kingdom from a patient with a recent history of hospitalization in India in 2011 [5]. Subsequently, NDM-5 isolates have been reported in China, India, Japan, Australia and Algeria [6,7,8,9,10] and are commonly carried by *Escherichia coli* strains, and the majority of the latter have been recovered from human patients.

Colistin is a representative member of the family of cationic polypeptide antibiotics and is generally considered the last line of antibiotic against MDR Gram-negative pathogens. In 2015, the *mcr-1*-bearing plasmid, which confers resistance to colistin, was first reported [11], and this rapidly developed into another severe challenge for human health. In our previous study, we identified a hybrid IncX3-X4 plasmid (pCQ02-121) that co-harbored *bla*_NDM-5_ and *mcr-1* in *E. coli* strain CQ02-121 isolated from a cat in Chongqing, China [12]. This hybrid plasmid was highly stable in both the original clinical isolate and corresponding transconjugants even in the absence of antibiotic selective pressure. This indicates that the strains of this type placed under selective pressures imposed by clinical treatments would most likely further increase the dissemination of the plasmid. Considering the close bond between companion animals and humans, it is likely that such a plasmid could be transferred to humans. Therefore, enhanced surveillance efforts are warranted to monitor the spread of these resistance elements.

In the current study, we investigated the prevalence of NDM-5-producing *E. coli* in companion animals in China and in an attempt to understand the dissemination of the IncX3-*bla*_NDM-5_-IncX4-*mcr-1* hybrid plasmid that was characterized in our previous study.

## 2. Materials and Methods

### 2.1. Bacterial Isolation and Species Identification

**Figure 1:**
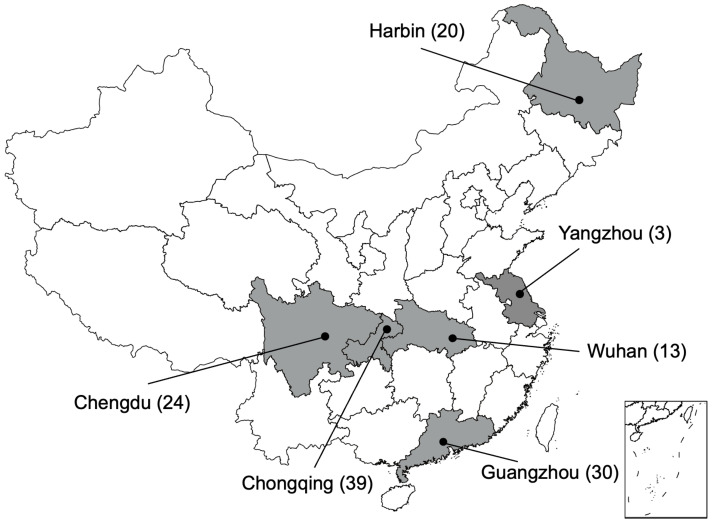
Map of sampling sites. The black dots represent the cities that the veterinary clinics were located in. The number of samples are marked after the name of cities.

From July to November 2015, a total of 129 rectal swabs of healthy or diseased dogs and cats were collected from veterinary clinics in six different cities including Harbin, Yangzhou, Chongqing, Wuhan, Chengdu and Guangzhou, in China (Figure 1). The information about the dogs and cats, including their age, gender, species, health status, reasons to go to the veterinarian was investigated (Table S3). All of the swabs were directly streaked onto MacConkey agar plates containing 1 mg/L meropenem and were incubated overnight at 37 °C; then, the agars with isolates were transferred to our lab within 24 h at low temperature. Colonies of different morphologies were picked and re-streaked for further identification. Bacterial species were identified by MALDI-TOF-MS (Matrix-Assisted Laser Desorption/Ionization Time of Flight Mass Spectrometry, Shimadzu-Biotech Corp., Kyoto, Japan).

### 2.2. AST and Detection of Carbapenemase-Encoding Genes

Antimicrobial susceptibility testing (AST) includes cefotaxime, ceftazidime, cefoxitin, meropenem, ertapenem, imipenem, aztreonam, amikacin, gentamicin, tobramycin, ciprofloxacin, tetracycline, tigecycline, fosfomycin, sulfamethoxazole–trimethoprim, colistin. The minimal inhibitory concentration (MIC) of these antimicrobial agents was determined by both agar dilution and micro-broth dilution based on the guideline of the Clinical and Laboratory Standards Institute (CLSI, 2015: M100-S28). *E. coli* American Type Culture Collection (ATCC) 25922 served as the quality control strain. Bacterial colonies were screened for carbapenemase production using the Carba NP test [13], and positive isolates were further screened for the presence of *bla*_KPC_, *bla*_IMP_, *bla*_VIM_, *bla*_OXA-48_ and *bla*_NDM_ (Table S2) by polymerase chain reaction (PCR) amplification and amplicon sequencing [14].

### 2.3. Molecular Typing, Plasmid Analysis and DNA Sequencing

Genetic relationships between the *bla*_NDM-5_-positive *E. coli* isolates were evaluated by pulsed-field gel electrophoresis (PFGE) following digestion of *Xba*I [15]. Comparisons of PFGE patterns were analyzed using BioNumerics software (Applied Maths, Sint-Martens-Latem, Belgium). Multi-locus sequence typing (MLST) was performed using the protocol specified by the *E. coli* MLST web site [16].

Conjugation and electroporation were used to access transferability of resistant determinants using *E. coli* strain EC600^Sm^ as the recipient [17]. Transconjugants were selected on MacConkey agar plates supplemented with meropenem and streptomycin and confirmed by both AST (as described in 2.4. Molecular Typing) and PCR amplification. Plasmid analysis of the transconjugants was carried out by DNA linearization with S1 nuclease followed by PFGE [18]. Plasmid DNA from CQ6-1T was sequenced using the HiSeq 2000 (Illumina) technology. Gene annotation and prediction were performed using RAST (Rapid Annotation using Subsystem Technology) and BLAST (Basic Local Alignment Search Tool) [19]. The complete sequence of pCQ61-NDM was taken as the reference sequence for PCR mapping of the *bla*_NDM-5_ genetic environment.

## 3. Results

### 3.1. Bacterial Strains and Antimicrobial Susceptibility Testing (AST)

**Figure 2:**
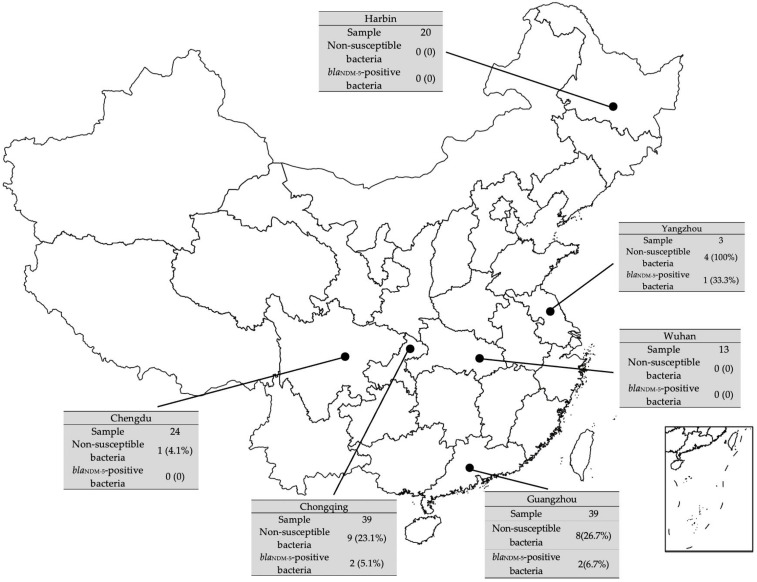
The detection rate of *bla*_NDM-5_-carrying *E. coli*. The black dots represent the cities that the veterinary clinics are located in. The isolation rate of carbapenems non-susceptible bacteria and detection rate of *bla*_NDM_-positive *E. coli* isolates are tagged as well.

**Figure 3:**
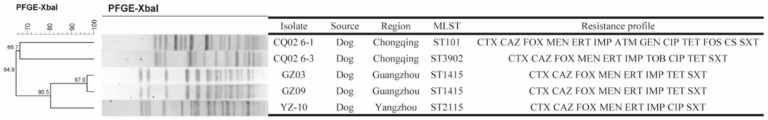
Clonal relationship, plasmid characteristics and antibiotic resistance phenotype of the five *bla*_NDM-5_-positive *E. coli* isolates. Dendrograms based on *Xba*I-restriction patterns of *E. coli* isolates producing NDM-5. The number on the left represents the similarity between corresponding strains. *E. coli* isolates showing similarities of <85% were considered to be unrelated. CTX, cefotaxime; CAZ, ceftazidime; FOX, cefoxitin; MEM, meropenem; ERT, ertapenem; IMP, imipenem; ATM, aztreonam; GEN, gentamicin; CIP, ciprofloxacin; TET, tetracycline; FOS, fosfomycin; CS, colistin; SXT, sulfamethoxazole–trimethoprim.

We investigated six cities located in northern, central and southern China. The isolation rate for meropenem-resistant isolates was 17.05% (22/129 swab samples), and the detection rate of *bla*_NDM-5_-carrying *E. coli* was 3.88% (five isolates), all from companion animals. The 5 *bla*_NDM-5_-positive *E. coli* strains were recovered from three different cities, Yangzhou (strain YZ-10), Chongqing (strains CQ6-1 and CQ6-3) and Guangzhou (strains GZ03 and GZ09), respectively (Figure 2). These five isolates harbored the *bla*_NDM-5_ allele that exhibited 100% nucleotide identity to the same gene from *E. coli* EC405 that was confirmed using CarbaNP tests and PCR amplification. AST indicated that all five isolates were resistant to cephalosporins, carbapenems and co-trimoxazole but were susceptible to amikacin and tigecycline (Figure 3, Table S1). Notably, strain CQ6-1 was also resistant to aztreonam, gentamicin and colistin, and the presence of *mcr-1* was identified simultaneously. These strains tested negative for the presence of *bla*_KPC_, *bla*_VIM_, *bla*_IMP_ and *bla*_OXA_.

### 3.2. Genetic Relatedness of Isolates

A multi-locus sequence typing (MLST) analysis indicated that these five *bla*_NDM-5_-harboring *E. coli* isolates comprised four kinds of sequence types: ST101 and ST3902 from Chongqing, ST1415 from Guangzhou and ST2115 from Yangzhou. PFGE also revealed that GZ03 and GZ09 were clonally related, and the others belonged to different clusters. Notably, CQ6-1 and CQ6-3 were isolated from the same dog but belonged to different STs (Figure 3).

### 3.3. Characteristics of the Bla_NDM-5_-Carrying Plasmids

Conjugation experiments coupled with PCR tests indicated that the *bla*_NDM-5_-carrying plasmids were able to be transferred to the strain EC600^Sm^ except for CQ6-3, which was obtained via electroporation of the plasmid. In strain CQ6-1, the *mcr-1* gene was mobilized and co-transferred by conjugation to EC600^Sm^. Transconjugants were resistant to the β-lactams meropenem, imipenem, ertapenem and cephalosporins but not to aztreonam. S1 nuclease-PFGE analysis of the transconjugants and the transformant revealed that all *bla*_NDM-5_ genes were located on plasmids of the same approximate size 46 kb (Figure S1). PCR-based replicon typing confirmed that all these *bla*_NDM-5_-encoding plasmids were type IncX3 (pNDM5-IncX3) and their genetic contexts were >99.99% identical to that of pNDM_IncX3, taken from *Klebsiella pneumoniae* in China (GenBank Acc. no. KU761328). The *bla*_NDM-5_ genes were adjacent to an upstream truncated IS*Aba125* that was interrupted by insertion of IS5, and the genes *ble*, *trpF*, *dsbC* and a remnant of *ctuA1*, truncated by the insertion of IS*26*, were located immediately adjacent downstream.

**Figure 4:**
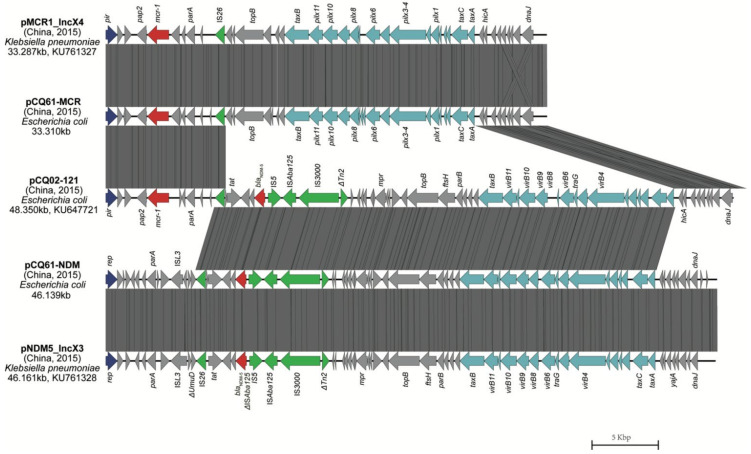
Linear sequence comparisons of pCQ61-NDM and pCQ61-MCR with other plasmids, including pMCR1_IncX4 (GenBank Acc. no. KU761327), pNDM5_IncX3 (GenBank Acc. no. KU761328) and from our previous study pCQ02-121 (GenBank Acc. no. KU647721). Boxed arrows represent the position and transcriptional direction of ORFs (open reading frames). Regions of >99% identity are marked by grey shading. Dark blue represents replication-associated genes, and light blue represents genes associated with the *pil*, *tra* and *vir* loci. ARGs are colored red; insertion sequences are colored green.

The transconjugant CQ6-1T contained not only pNDM5-IncX3 but also an *mcr-1*-encoding IncX4 plasmid (pCQ61-MCR) indicating the co-transfer of both *bla*_NDM-5_ and *mcr-1* carrying plasmids. pCQ61-MCR was 33,310 bp in length and had 100% BLAST query coverage and 99.9% nucleotide identity to plasmid pMCR1-IncX4 (GenBank Acc. no. KU761327). Since strain CQ6-1 was isolated from the same area as the previously reported strain CQ02-121 that possessed an IncX3-IncX4 hybrid plasmid as well as *mcr-1*, as expected, *bla*_NDM-5_ and *mcr-1* align between the CQ02-121 hybrid with the pCQ61-NDM and pCQ61-MCR plasmids in CQ6-1 (Figure 4).

## 4. Discussion

To the best of our knowledge, this is the first study of NDM-5-producing *Enterobacteriaceae* in companion animals in China. Since its first identification in the UK, *bla*_NDM-5_ has been detected in clinical isolates belonging to many STs from Asia, Africa, Europe, Australia and the United States [6,7,8,20,21,22,23,24,25,26]. In contrast, the prevalence of NDM isolates from companion animals has been sporadic [27,28,29,30]. The first four cases were from dogs in Algeria, Finland, South Korea and the United States, and the fifth was an ST156 isolate from a cat. In the current study, we found that in six cities, the prevalence of resistant bacteria was 17% with *bla*_NDM-5_-harboring *E. coli* at 3.86% overall from companion animals in China. This rate of *bla*_NDM-5_-positive isolates in companion animals was closer to human clinical data [31,32]. The presence of these isolates in companion animals has become a potential risk and that the transmission of carbapenem and colistin resistance has been shared between humans and companion animals.

MLST analysis revealed that five *bla*_NDM-5_-positive *E. coli* belonged to four STs among which an epidemic clone ST101 was in isolate CQ6-1 from Chongqing. *E. coli* ST101 differs from the ST131 isolates that have been previously associated with the clonal dissemination of extended spectrum β-lactamases. At the initial epidemic stage of *bla*_NDM_, ST101 was the most prevalent ST associated with *bla*_NDM-1_ in a collection of isolates from England and Pakistan, as well as Australia, Germany, Denmark and Bulgaria [3,33,34,35]. NDM-5-producing *E. coli* have also been typically described as ST101 members [36], although two novel variants NDM-8 [37] and NDM-13 [38] were both initially detected in *E. coli* ST101. All the evidence indicates that ST101 has become an important reservoir of *bla*_NDM-1_ and its variants.

It is noteworthy that we found two NDM-5-producing strains (CQ6-1 and CQ6-3) that were isolated from the same dog but possessed different STs and PFGE patterns and shared an identical genetic environment (Figure 3). The horizontal gene transfer of multiple *bla*_NDM-5_-carrying *E. coli* strains in the dog was most likely responsible for this phenomenon. In addition, isolate CQ6-1 was resistant to both carbapenems and colistin. In agreement with the resistance phenotype, this strain contained both *bla*_NDM-5_ and *mcr-1*, and the *bla*_NDM-5_-encoding plasmid was almost identical to the IncX3 plasmid pNDM5-IncX3 and *mcr-1*-encoding plasmid pCQ61-MCR, which showed nearly the same nucleotide sequence as IncX4 plasmid pMCR1-IncX4 from *K. pneumoniae* [39]. In our previous study, the isolate CQ02-121 harbored *bla*_NDM-5_ and *mcr-1* on a transferable IncX3-X4 hybrid plasmid [12]. Strain CQ6-1 was isolated from the same veterinary clinic in Chongqing, China as CQ02-121 but contained *bla*_NDM-5_ and *mcr-1* on separate IncX3 and IncX4 plasmids, demonstrating that *bla*_NDM-5_ and *mcr-1* plasmids may evolve by hybridization or by splitting to transfer the resistance genes under antibiotic selective pressure. Based on existing reports and the present study, the most likely scenario for this is that the initial strains harboring *bla*_NDM-5_ or *mcr-1* existed in human and animals and under selective antibiotic pressure moved between human and animal hosts and further formed strains that co-harbored *bla*_NDM-5_ and *mcr-1*. This type of transmission contributes to ARG dissemination and could risk human health. We therefore performed a Web of Science database search for the presence of NDM and MCR co-producing strains. The vast majority have been detected in China, suggesting that dissemination of such strains in China has been extremely rapid and may eventually facilitate transmission to other parts of the world through traffic and trade.

IncX3 plasmids are narrow-host-range plasmids in the *Enterobacteriaceae* and are a common vehicle for *bla*_NDM_ transmission. Plasmids that contain *bla*_NDM_ alleles and especially the *bla*_NDM-4_-like variants (*bla*_NDM-4_, *bla*_NDM-5_, *bla*_NDM-7_ and *bla*_NDM-8_) were found to have nearly identical backbones regardless of the original strain background [40,41]. Since the discovery of the *bla*_NDM-4_-like variants, *bla*_NDM-4_ and *bla*_NDM-5_ have been identified in *Enterobacteriaceae* from humans, milk, sewage, dogs, fish and ducks across five continents. The genetic contexts for the placement of *bla*_NDM_ within the plasmids from these strains were nearly identical, suggesting that the IncX3 plasmid carrying *bla*_NDM-1/4/5/7_ had a common plasmid ancestor.

## 5. Conclusions

In conclusion, this study characterized *NDM-5*-producing *E. coli* from companion animals in China. The *bla*_NDM-5_ gene was located on a 46 kb transmissible IncX3 plasmid in all five isolates, and the genetic context of the IncX3 plasmids was nearly identical to that of the *K. pneumoniae* plasmid pNDM5-IncX3 previously reported from China [39]. Importantly, *bla*_NDM-5_ and *mcr-1* were able to co-transfer to the recipient, suggesting that plasmid transmissibility contributes to the dissemination of *bla*_NDM-5_ in companion animals. Companion animals have close relationships with humans and may play an essential role in the transmission of carbapenemase-producing isolates, as well as act as a reservoir of important resistance genes. Further monitoring and study might be necessary to survey the dissemination of carbapenemase-producing pathogens and to investigate the prevalence of carbapenem and colistin resistance.

## Data Availability

The data will be available with the corresponding author upon request.

## Supplementary Materials

The following supporting information can be downloaded at: https://www.mdpi.com/article/10.3390/ani12101310/s1, Figure S1: Localization of *bla*_NDM-5_ carrying plasmids by S1 nuclease PFGE and Southern hybridization. (a) S1-PFGE gel of the transconjugants/transformant. (b) Southern hybridization conducted with a probe specific for *bla*_NDM_. Line 1, GZ-03-T; Line 2, GZ-09-T; Line 3, CQ-61-T; Line 4, CQ-63-T; Line 5, CQ02-121T; Line 6, YZ-10-T. Table S1: MICs of sixteen antimicrobials agents against NDM-5-positive isolates and transconjugants. Table S2: Primer sequences for carbapenem resistance genes. Table S3: Samples information.
