# Supplementary material for: NDM-5-Producing Escherichia coli Co-Harboring mcr-1 Gene in Companion Animals in China
Source: Animals (Basel). 2022 May 20;12(10):1310. doi: 10.3390/ani12101310 (PMC9137672; doi:10.3390/ani12101310)
Supplement: Supplementary file 1 [file animals-12-01310-s001.zip › Tables S1&S2.pdf]

Table S1. MICs of sixteen antimicrobials agents against NDM-5-positive isolates and transconjugants.

Strains	MICs (mg/L)															
	**CTX**	**CAZ**	**FOX**	**MEM**	**ERT**	**IMP**	**ATM**	**AMK**	**GEN**	**TOB**	**CIP**	**TET**	**TGC**	**FOS**	**SXT**	**CS**
CQ02 6-1	>256	>256	>256	16	>64	>64	>256	2	64	8	>256	256	1	128	>320	4
CQ02 6-3	256	>256	>256	64	>64	>64	32	4	16	64	256	128	0.5	4	>320	0.25
GZ03	256	>256	>128	4	64	64	0.5	4	1	16	2	64	0.5	4	>320	0.5
GZ09	256	>256	>128	4	32	>64	0.25	2	1	8	2	64	0.5	2	>320	0. 5
YZ-10	128	>256	>256	16	>64	32	4	2	0.5	0.5	32	8	0.5	8	>320	0.5
Transconjugants																
CQ02 6-1T	128	128	>128	4	>64	32	0.5	2	2	2	0.03	2	0.5	1	10	4
CQ02 6-3T	128	128	128	8	64	32	0.25	2	2	2	0.03	2	0.5	2	10	0.25
GZ03T	64	256	>128	1	64	32	0.0625	2	2	1	0.008	1	0.015	2	10	0.125
GZ09T	64	256	>128	1	>64	64	0.125	2	2	1	0.015	1	0.0625	2	10	0.125
YZ-10T	256	128	>128	16	>64	16	0.25	2	2	2	0.03	1	0.0625	2	10	0.125

CTX, cefotaxime; CAZ, ceftazidime; FOX, cefoxitin; MEM, meropenem; ERT, ertapenem; IMP, imipenem; ATM, aztreonam; AMK, amikacin; GEN, gentamicin; TOB, tobramycin; CIP, ciprofloxacin; TET, tetracycline; TGC, tigecycline; FOS, fosfomycin; SXT, sulfamethoxazole–trimethoprim, CS, colistin.

Table S2. Primer sequences for carbapenem resistance genes.

Target genes	Primers（5'-3'）	Length（bp）	Tm（℃）
*bla*NDM	GGTTTGGCGATCTGGTTTTC<br>CGGAATGGCTCATCACGATC	621	56.1
*bla*KPC	CGTCTAGTTCTGCTGTCTTG<br>CTTGTCATCCTTGTTAGGCG	798	58.2
*bla*OXA-48	GCGTGGTTAAGGATGAACAC<br>CATCAAGTTCAACCCAACCG	438	57.7
*bla*IMP	GGAATAGAGTGGCTTAAYTCTC<br>GGTTTAAYAAAACAACCACC	232	54.6
*bla*VIM	GATGGTGTTTGGTCGCATA<br>CGAATGCGCAGCACCAG	390	56.3
