# Supplementary figures and images for: NDM-5-Producing Escherichia coli Co-Harboring mcr-1 Gene in Companion Animals in China
Source: Animals (Basel). 2022 May 20;12(10):1310. doi: 10.3390/ani12101310 (PMC9137672; doi:10.3390/ani12101310)

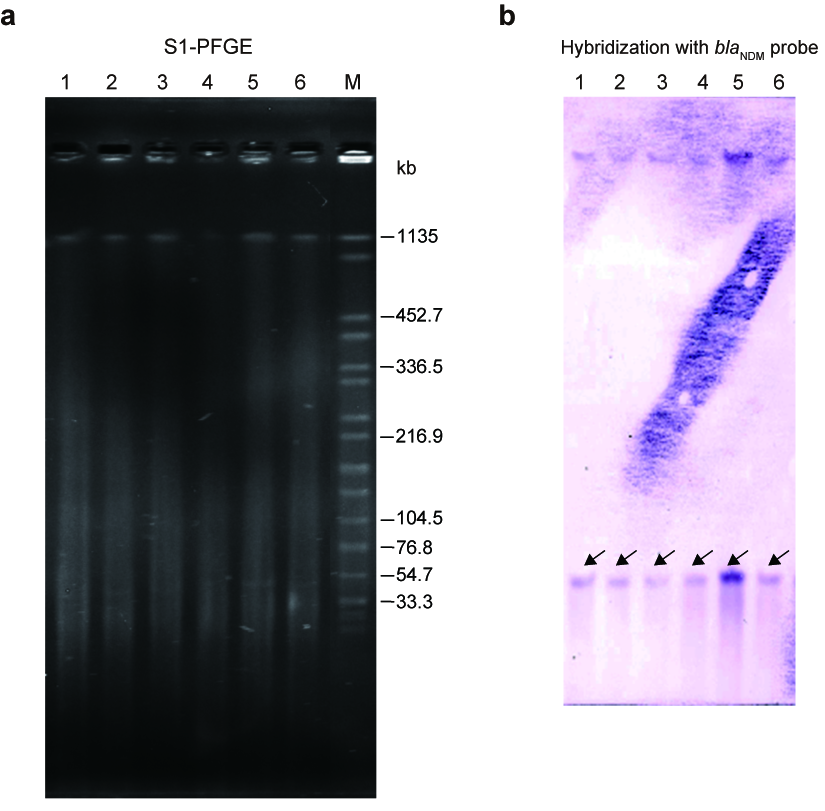

Supplement: Supplementary file 1 [file animals-12-01310-s001.zip › Figure S1.tif]
